# Moving our care home: A qualitative study of the views and experiences of residents, relatives and staff

**DOI:** 10.1111/opn.12466

**Published:** 2022-04-18

**Authors:** Kinda Ibrahim, Sophie Baron, Judith Lathlean, Jackie Bridges, Nuala McGrath, Helen C. Roberts

**Affiliations:** ^1^ 7423 Academic Geriatric Medicine Faculty of Medicine University of Southampton Southampton UK; ^2^ 7423 NIHR Applied Research Collaboration Wessex University of Southampton Southampton UK; ^3^ Faculty of Environmental and Life Sciences School of Health Sciences University of Southampton Southampton UK; ^4^ 7423 Primary Care, Population Sciences and Medical Education Faculty of Medicine University of Southampton Southampton UK; ^5^ University of Southampton and University Hospital Southampton NHS FT Southampton UK; ^6^ NIHR Southampton Biomedical Research Centre (BRC) Southampton UK

**Keywords:** care home, older, qualitative, relocation, resident

## Abstract

**Introduction:**

Involuntary relocation when care homes close can be detrimental to residents’ health and well‐being and is associated with increased mortality. There is little formal evidence to support whether planning can mitigate the impact of such moves. This study aimed to understand the experiences of a whole care home relocation where staff and residents relocated together using existing published guidance.

**Methods:**

A longitudinal qualitative research study using individual face‐to‐face semi‐structured interviews was conducted between August 2018 and August 2019. Baseline interviews were conducted 6–8 weeks after relocation with follow‐up interviews 10–12 months later. Interviews were recorded, transcribed and analysed using framework analysis.

**Results:**

27 interviews were conducted; 19 baseline interviews (4 residents, 7 family members, 8 staff) and 8 follow‐up interviews (2 residents, 2 family members, 4 staff). Participants’ feelings about the relocation were mixed: some reported apprehension before the move but others excitement. Residents and families felt variably involved in planning the move, whereas staff expressed lack of involvement. Time, family support and continuity of care helped participants settle in. The new environment shaped participants’ experiences and abilities to adjust, especially the lack of a homely feeling with the new home, the larger size and changes in staff organisation and management.

**Conclusions:**

Despite implementation of existing guidance, relocation was still challenging for residents, staff and family members. Future relocations should increase involvement of staff in the planning and design of the home; offer continuous support to those involved; and ensure continuity of care and management style.

## 1 INTRODUCTION

Increasingly care home residents face the challenge of relocation due to care home closure. A 2019 survey by the UK Association of Directors of Adult Social Services (ADASS) reported that 115 care home providers closed or ceased to trade across 72 councils over 6 months periods in 2019 and more than 7,000 residents had been relocated (ADASS, 2019). While care homes may close for a variety of reasons including financial and staff recruitment pressures, the UK Health and Social Care Act 2008 (Regulated Activities) Regulations in 2014 led to stricter building regulations for UK care homes, for example, premises should be easily accessible to service users and each resident should have an en‐suite bathroom (CQC, 2014). Many established care homes in older buildings that did not satisfy these new regulations have had to close or move to new premises. Similar patterns of care home closures due to financial losses and multiple sanctions levied against the facility from state regulators were reported internationally (Capezuti et al., 2006; Castle, 2005; Falk et al., 2011).

Care home relocation can be categorised into the following: preference relocation where residents have exercised choice; strategic relocation where the resident pre‐empts relocation and moves beforehand; and involuntary relocation where the decision is made by others on the resident's behalf (Kleit & Galvez, 2011). The European Court of Human Rights recognised the negative effects of care home relocation, particularly on residents who are older, male, living with cognitive, mental or sensory impairment, or with reduced mobility (Jolley et al., 2011; Robinson et al., 2013). They identified elements of a move which could produce the greatest stress including sudden or unplanned moves, failure to assess and meet medical and psycho‐social needs, discontinuity of care, lack of consultation with residents and families, and lack of information and explanation of rights and options. Reluctant or passive care home relocation is associated with a perceived lack of choice in the move, loss of autonomy, feeling sad due to the closure, and increased mortality (Holder & Jolley, 2012; Leyland et al., 2014; Smith & Crome, 2000).

Nurses and care assistants are the main professional groups affected by care home closures. They can support residents through the relocation but may experience significant personal implications. One study interviewed 63 long‐term care workers who relocated, and identified loss of relationships between staff, a change in roles that they were expected to perform and an overall increase in stress (Canham et al., 2018).

Jolley and colleagues produced detailed recommendations in 2011 for care home managers before, during and after the relocation. These include information sharing, counselling for staff and residents, comprehensive medical checks, familiarisation visits, detailed handover and continuity of care (Jolley et al., 2011). The first UK national guidance for care home closure in 2016 explained the role and responsibility of the organisations involved (Department of Health, 2016a, 2016b). These cover the importance of maintaining confidentiality, promoting autonomy, the assignment of co‐ordinators and the sensitive handling of communication throughout the process. Residents should be provided with the broadest range of options available and aided in decision‐making. Residents, family members and staff should be informed consistently with a detailed handover of each resident to their new care home, identification of those deemed at higher risk of the effects of relocation and a review of all residents within 4 weeks. However, current guidelines do not address the scenario of a whole care home relocation where staff and residents relocate together from a care home.

This research investigates a recent care home closure in England and the subsequent relocation of both residents and staff to a new building. The move was planned using the above published guidance for all stages of the relocation (Department of Health, 2016a, 2016b; Jolley et al., 2011). The aim was to understand the experiences of a whole care home relocation from the perspective of those involved including residents, family members and staff, over 1 year.

## 2 METHODS

### 2.1 Setting and care home relocation

Residents and staff moved from a 28‐bed private care home in an old building to a new, purpose built 64‐bed care home in the same village owned by the same care home organisation in August 2018. Preparations for the move based on published guidance included the following: early notification of the closure and move to residents, families and staff; regular communication with all those involved; offering residents and their families the opportunity to choose their room and its decoration from the building plan; and arranging familiarisation visits later in the construction. A few residents (5–6) were relocated each day over 5 days to focus on settling them in, with staff spread across the two care homes. Residents living in adjoining rooms in the old care home moved together to minimise disruption to other residents and family members, volunteers and staff helped pack and move belongings. In the new care home, a staff member escorted each resident to their new rooms which had welcoming cards and flowers. The same food menu was served in both care homes during the moving week only.

### 2.2 Study design and sample

Relocation is considered a process rather than a single event; therefore, a longitudinal follow‐up qualitative research study using semi‐structured interviews was conducted (Holland, 2007). Baseline interviews were conducted between September and October 2018 (6–8 weeks after relocation) with follow‐up interviews between June and August 2019 (10–12 months after the move). Purposive sampling for the baseline interviews was used to recruit a range of participants who included:

- Residents who had relocated from the old care home to the new care home and who had the capacity to understand the nature of the research and consent.
- Family members of residents who frequently visited the care home, including those of residents who were unable to be interviewed due to lack of mental capacity.
- Staff members from the old care home who moved to the new care home including members of the management team, nurses, healthcare assistants and administration and coordination team.

This study was given ethical approval by the University of Southampton, Faculty of Medicine Ethics Committee and Research Governance Office (ERGO Number: 42437).

### 2.3 Recruitment and data collection

The care home management team was asked to identify eligible residents and identified 12 residents who met the eligibility criteria. Residents were considered ineligible for interviews if they were very unwell or cognitively unable to engage in a conversation. All potentially eligible residents (*n* = 12) were then approached by the research team who explained the study and answered any question, and they were given at least 48 h to think, talk to family and then decide whether to take part or not. Four out of 12 (33%) approached agreed to take part in the study (three females and one male). Reasons for not taking part included the following: residents’ unwillingness to take part, for example, one resident was worried about the recording of the conversation, and family members feeling that their relatives were not well enough to participate. Eleven staff members who transferred from the original care home to the new care home were identified by the care home management team and were approached in person by the research team who explained the study. Eight female staff members agreed to participate (one from the management team, three from the administration and coordination team, two nurses, and two healthcare assistants). The management team sent out invitation letters to ten family members who visited the home regularly and were involved in the care of their relatives or lived locally, therefore, were aware of their relatives’ experience and able to potentially be interviewed (irrespective of residents’ eligibility); four replied and agreed to be interviewed. Three further family members were identified and approached by a researcher through regular visits to the care home. The seven family members were five daughters, one son and one daughter‐in law. The 19 baseline interviews took place 6–8 weeks post relocation to minimise recall bias.

Follow‐up interviews were conducted 10–12 months after relocation. The aim was to interview the same participants to understand whether and how their experience in adjusting to the new environment has changed over time. Eight of the 19 (follow‐up rate of 42%) agreed to be re‐interviewed. One resident had died and one declined, thus two of the four residents were approached again in person by the research team and were re‐interviewed. Four of the eight staff participants were on leave or no longer working in the new care home. The remaining staff were contacted by personal visits to the care home, by email and/or telephone, and four staff were re‐interviewed. The roles of staff members remained the same at 12 months follow‐up. The seven family members were sent letters or emails and two agreed to be re‐interviewed (one refused as their relative had died; one was contacted by email and phone with no reply; three had lost contact).

Three baseline semi‐structured interview guides were prepared for each of the stakeholders and all focused on exploring participants’ experiences of the closure of the old care home, the relocation to the new care home, and expectations of and early adjustment to the new environment. Follow‐up interviews were similar but focused on participants’ experiences and adjustments over time. All interviews took place in a private room in the new care home, as chosen by the participant, and were audio‐recorded.

### 2.4 Data analysis

All recordings were transcribed verbatim and destroyed after transcription. Transcripts were anonymised and any names or identifiable information were removed with each participant given an ID code. We kept a record of the participants name and contact details for follow‐up interviews which was accessed by a delegate member of the research team and was destroyed at the end of the follow‐up. Anonymised transcripts (electronic files) were stored on a password protected computer and the consent forms (hard copies) were stored in a locked filing cabinet in a secure office in our research unit and were accessible only by the research team.

Baseline interviews were analysed using the Framework method (Furber, 2010) by two authors (SB & KI). Using this method, a number of steps were followed in the analysis including the following: transcription, familiarisation with the data, coding, developing a working analytical framework, applying the analytical framework charting data into the framework matrix and interpreting data. A descriptive coding framework was developed from transcripts based on participants’ perceptions and experiences to highlight commonalities and differences using constant comparisons. The focus was on coding data that were the most significant and relevant to the research questions and the desire was to keep as close as possible to the accounts of participants. Coding followed an inductive approach and was data driven rather than based on a prior framework or driven by a theory. This helped establish the real impact of closure and relocation on people involved and explored practical lessons and recommendations.

The coding framework developed at baseline was then used by (JL) to analyse the follow‐up interviews to allow comparisons of any changes in experiences over time. However, the researchers remained open to new codes and concepts emerging in the follow‐up interviews. Quotes from interviews have been selected both to illustrate the data underpinning the emerging themes, and to present the range of data across the different participant groups.

### 2.5 Reflexivity and Trustworthiness

The research team consists of female academics with different levels of seniority (medical students, a postdoctoral researcher and professors). Two of these academics were trustees of the care home charitable foundation. Their experience and expertise were valuable to the design of the study. To minimise their biases, they were not involved directly in data collection and analysis. Three other members of the team with different seniority and age levels (a medical student, a postdoctoral researcher and a professor in health science) were involved in data collection and analysis. They were all familiar with the care home sector with some personal experience either from working in a care home or visiting friends and relatives in care homes. This helped them relate to the residents, enabled them to read body language alongside responses. This experience also assisted in the identification of participants who may have been unable to consent and helped prompt further discussion with staff as to their eligibility for the study. The involvement of the three researchers in the process of data collection and interpretation was key to minimise any personal bias and to ensure the trustworthiness of the findings. Researchers kept individual reflexive journals during data collection and analysis, and these were discussed and shared with other researchers during the regular research team meetings. In addition, we used data triangulation (i.e. collecting data from different group of participants—relatives, residents and staff—to ensure the whole picture of experiences of relocation was captured. In addition, a report of the main findings and recommendations for future relocations were shared with the care home organisation and the feedback from their senior management team confirmed that they recognised the validity of our findings. We also know that the care home organisation has rewritten their protocol for care home relocation in light of these findings.

## 3 FINDINGS

In total, 27 interviews were conducted; 19 baseline interviews (4 residents, 7 family members, 8 staff) and 8 follow‐up interviews (2 residents, 2 family members, 4 staff). The findings from the two sets of interviews are presented in relation to five themes: attitudes towards relocation, communication and involvement, environmental changes and impact, staff organisation and management, and adjustment and settling in.

### 3.1 Attitudes towards relocation

The attitudes of participants towards the closure and relocation to the new care home did not change markedly over time. In the baseline interviews, most participants were apprehensive about the relocation and what the new care home would be like. Many participants were sad about the closure of the old care home and described a very close‐knit community which felt like a family. The old building, although tired, seemed cosy and like a ‘normal’ home to residents and their families.

Staff members worried whether they would be able to keep their jobs, and some had expected to work in the old home until retirement. Relatives were concerned how residents would respond to the move:

Very worried, because you know I didn’t know how she would respond… you hear about the changes not being good for somebody who’s… not as mentally strong as they were before, and it was the unknown really, and because of her age and that, you know. (Family member)

Familiarisation visits to the new care home were arranged for staff, residents and their families. A large number of participants reported that they visited the new care home too early, and that it was hard to visualise what it was going to be like, as only parts were open and it was unfurnished:

No I’ve been…, months ago when they were building it, when… it was a very, very cold day, freezing it was, and we came in and had… nothing, it was just brick, they’d just done the inside, and we started waiting, oh it’s freezing, it was really cold. Everybody was complaining ‐ why have we come here’. (Resident)

However, there was also excitement about the new care home from a few participants who reported that seeing plans and visiting the new home helped them envisage the care home and hoped that relocating would be a good experience:

I was looking forward to it, because [the old care home] was lovely, but like anything, sometimes you need to move to improve. (Staff member)

In the follow‐up interviews, one resident who had lived in the old home for many years remained unhappy about leaving somewhere that felt so ‘homely’ and where she knew most of the other residents and staff.

Conversely, the other resident in the follow‐up interviews had only lived in the old home for a few months and could only see advantages in the move; she now had a bigger room with en‐suite facilities, and she had not had so long to form relationships with the other residents in the previous home.

### 3.2 Communication and involvement

All participants in the baseline interviews reported that they had been notified of the closure of the old care home at a number of times and through letters and meetings with the care home senior management team. Participants felt that the information given was sometimes limited, and changed over time.

And the difficulty with the process…is that the information changed, over a period of time, before we moved, so what was promised at one point was different at another meeting. (Family member)

Some of us had said one thing and then later were told to do something else. And that happened quite a lot. (Staff member)

In the baseline interviews, some residents and family members reported sufficient involvement in the process while others felt less involved or not involved at all.

We were being involved, told as relatives, you know, the days that the different residents were going…yeah I felt well informed prior to the move, they seemed very organised and wanting to make it as smooth a transition as possible. (Family member)

Many staff and family members suggested in the baseline interviews that residents did not have much choice over whether they relocated to the new home or not, saying ‘*It's just like us moving home*, *but we've got a choice when we move home*, *they haven't*’. However, involvement of residents was encouraged as most had some choice in the location of their bedroom and the bedroom's colour scheme in the new care home, as well as artwork for the communal areas.

Staff members felt they were not invited to input and expressed a desire to be more engaged in planning the relocation and the design of the care home. Each staff member had an individual consultation with senior management prior to the relocation but discussions focused on future career roles and new job terms were seen by some to be one‐sided and inflexible:

We had what we class as interviews, cause that’s what it was like, we were being interviewed and weren’t allowed to give any input. We were just told what was happening. It wasn’t up for discussion. (Staff member)

In the follow‐up interviews, some staff and family members felt that lack of involvement in the planning had indeed proved to be an issue that could have been avoided in first place. They identified difficulties with using equipment with a lot of furniture in the bedrooms, and with accommodating enough wheelchairs in the dining area.

### 3.3 Environmental changes and impact

#### 3.3.1 The impact of size and design

In the baseline interviews, many participants described the new home as a ‘hotel’, rather than a home, based on the large size of the building and the design features. The size and layout of the building was mainly seen to impact both residents and staff negatively, although one staff member felt it was positive because the residents had more space.

Many participants initially found the building confusing to navigate, with a lack of signage, particularly affecting staff members. Staff workload had increased as a result of providing care over a larger area and the logistics of running activities were impacted by difficulties in getting residents to the activity hub downstairs:

But it’s just like when having a quiz in the morning, there’s a lot of them that like to do it but you physically can’t get round to everybody and bring everybody down. (Staff member)

The layout of the dining room was frequently brought up by participants as a downside of the home environment. This had an effect on the dining experience and social interaction, with the room being noisy and cramped with difficulty fitting wheelchairs around the tables:

There's barely enough room for them…if they all came out, there isn't enough room. (Family member)

In the follow‐up interviews, most participants were finding their way around the home but the large size, the new division into four households and certain design features such as the dining areas were still causing problems for some residents.

My [relative] won’t come out of her room. Unless I come in for lunch she will not come out because she finds it too confusing. (Family member)

Since coming here I have found it more difficult getting from my room to the [dining area]. [The home] is so big – the corridors are so long and confusing; in [the old home] we had one main dining room and not so far to go. So I choose to stay mostly in my room. (Resident)

However, some staff members felt that the new care home layout was better:

There’s more choice of where they can go and what they can do, because they’re not confined just to their room, or to one room there’s lots of different places they can go – which is nice I think. Cos some people want to go and sit and chat in a quiet room; some people want the television room, but there’s so many different places they can go and just be, do what they want to do. (Staff member)

#### 3.3.2 The co‐location of residents with dementia

In the old home, the structure and facilities meant that residents who had progressive dementia could not continue to be cared for. This was not the case in the new home which had dedicated households for residents with dementia. Most felt that this was desirable in theory, especially as residents who became in need of more support as a result of dementia could be accommodated in the same home:

The dementia side is such a nice idea, having it all in one place where they can be in a safe environment and they've still got the good nursing care. (Staff member)

However, one resident suggested that they were less sure, since it might be difficult to staff the area for residents with dementia. Further, one family member believed that having residents with early dementia integrating with those who were ‘*completely healthy*, *from a mental health point of view*’, as was the case in the previous home, was more beneficial.

### 3.4 Staff organisation and management

In the baseline interviews, staff and family members commented on the management restructuring that occurred after the relocation, with a change in the manager and additional deputy mangers. Management style was also perceived to have changed and some staff members as a result felt less supported.

After the relocation, there was a change in staff hours with shifts starting and ending at new times. This was reported as having a negative impact on staff. Residents were also affected as they were getting up later than previously which meant that some missed out on morning activities:

All the morning procedures now encroach on the activities, so sometimes they can’t get down for the activities, ‘cause that starts at half past 10. So they’re maybe not getting as involved as they should do. (Staff member)

There was a perception among residents and family members that the increases in staffing were not sufficient for the total increase in number of residents. The workload and the new changes impacted negatively on staff morale, both new and old staff, leading to resignations:

I do worry because the old team are so stressed that it rubs off on the new team and then the new team feel quite disheartened as well and I would say team morale is not good at the moment. (Staff member)

Both family members and residents in the baseline and follow‐up stages raised concerns that new staff did not know the residents well. Family members felt less informed about their relative's care and they considered that the promised continuity of staff for residents after the move did not happen. The established staff members explained that new staff, many of whom were from an agency, increased their own workload, because they had to invest time in training them, as well as complicating communication.

Further, the difficulty of providing equity of care and access to facilities such as outings and activities was highlighted—by some staff and family members—because of the size of the new home and the numbers of staff needed to fetch, chaperone or support residents. For example, activity co‐ordinators were attempting to provide such opportunities for much larger numbers of residents and were thus ‘*having to spread themselves thinly*’.

### 3.5 Adjustment and settling in

In the baseline interviews, some residents reported adjusting well, whereas others were still struggling. Family support, continuity of care and involvement in activities were potential factors which could have a positive impact on residents. Participants mentioned how time was essential to allow residents and staff to adjust well to the new environment:

Well it is taking time. I think that’s partly because of the new staff and everybody having to get used to it. And we’re all settling in, it will get better as time goes on. (Resident)

Nevertheless, in the follow‐up interviews, settling in was still difficult for some while others had managed to adjust. For example:

A couple of [residents] have settled in really quickly; for some of them it did take a while to settle in, especially as we were such a small home and now we’re such a big home. They were used to having 7–10 residents in one area and now, all of a sudden they’re 16, so, yes, and they struggled for a month over the size. But as did we all [including staff] really. (Staff member)

Reasons for failing to settle in, which were reported in the baseline and follow‐up interviews, included the size of the new home, new and different staff, the lack of a homely feel, lack of continuity of care and missing the old care home. In the follow‐up interviews, one family member described how her relative was still not coping well after 1 year.

It’s too big. I opted for a small home for [my relative] and this is three times the size of [the old home]. There’s no continuity with the carers here whatsoever; they are moved around constantly. Two days is maximum here anywhere, any household, so they don’t get to know the residents. Whereas in the old home, you had the same carers, very little movement of staff leaving and they knew the residents, their quirks and everything. (Family member)

## 4 DISCUSSION

The aim of this study was to understand and report residents’, family members’ and staff experiences over time of a planned whole care home relocation and to use these experiences to make recommendations to augment current guidance. This guidance was followed yet feelings were mixed about the relocation with some reporting apprehension in advance of the move and others excitement. Similarly, some people felt informed about and involved in planning the move, while others did not. The process of relocation and settling in impacted different people in different ways, with the passage of time seen by staff to aid residents in in this respect. Some features about the new environment shaped people's experiences and perceptions following the move, in particular the physical size and layout, the co‐location of residents with dementia and staff organisation and management. Several recommendations have been drawn from this research to aid future relocations (Box 1).

BOX 1 **Recommendations for group relocation of care home residents and staff**

**Pre‐relocation**

‐ Ensure ongoing appropriate communication about the closure and relocation to all those involved including residents, staff and family members (e.g. distribution of information sheets, newsletters and meeting minutes).
‐ Ensure information provided is regular and consistent to avoid any disappointment or dissatisfaction.
‐ Involve staff with different seniority levels (healthcare assistants, nurses, activity coordinators and more senior staff) as well as residents and their families in decisions regarding the move and the building design.
‐ Arrange the familiarisation visits for residents and their families to the new care home at an appropriate stage of building development, ideally when the development is nearly ready.
‐ Offer staff members the chance to spend time in the new care home to get familiar with the new building and navigate the space before moving residents.
‐ Offer residents and relatives the chance to visit the new care home to assess where their room will be in relation to other facilities.
‐ Encourage residents to choose their rooms near to their friends to maintain friendships and relationships.
‐ Offer emotional and psychological support to residents, their families and staff not only to address any concerns about the move but also to manage feelings around the move and its necessity.

**Post‐relocation**

‐ Staff rotas should maximise continuity of care for residents during the settling in period.
‐ Ensure that residents and their families have one point of contact to speak to throughout the whole process.
‐ Provide orientation within the new environment for residents and their families as well as new staff.
‐ Ensure specific staff members role is to help settle the person and their family into the new home. Their role should include meeting the person and family prior to the move, explaining the procedure, accompanying the person and family to the new home and orienting them to the home, sitting with them at meals and activity programmes for the first few weeks, introducing them to staff, other residents and families, providing support and information and checking that needs and preferences are being met.
‐ Provide counselling opportunities for individual residents, family members and staff to discuss and come to terms with the experience.
‐ Identify residents as well as staff who are struggling to settle into the new environment and offer them extra emotional and psychological support.
‐ Offer staff access to a dedicated member of the team to help them navigate the whole process, providing extra help, orientation, emotional support etc. until the staff feel settled in the new environment.
‐ Ensure a continuation in management and management style to minimise disruption to staff.
‐ Ensure staff working patterns and shifts remain similar to those in the old care home.
‐ Ensure sufficient staffing levels.
‐ Organise team building exercises to encourage integration and teamwork between established and new staff members and maintain high morals.

Facilitate a safe environment in which residents, family members and staff can express their worries and preferences freely to inform actions and changes.

These findings paint a picture over time and are important as senior managers worked hard to apply the known guidance on care home relocation. We report some issues with communications, inconsistent information and involvement in decision‐making regarding the closure and relocation which have been highlighted in the literature (Glasby et al., 2019) and Box 1 includes suggestions to address these important issues. The original care home was described as homely with a tight‐knit community, and yet had become increasingly unfit for purpose in the light of modern regulatory standards for care of people with physical and/or cognitive impairments. While the move enabled these standards to be more easily met, the process of transition disrupted the sense of community and made some people feel left out of important decisions, while the destination physical environment felt less homely for some. This potential tension between the requirements for state‐of‐the‐art buildings, complying with contemporary regulations, compared with the emphasis of residents and their families on ‘homeliness’ has been reported elsewhere in the literature (Peace & Holland, 2001). Continuing to feel at home when relocating care home is a very important need to be addressed whether the care home is newly built or not.

Sarnio and colleagues explored the meaning of ‘at‐homeness’ in old age and linked it to two interrelated facets: being oneself and being connected (Saarnio et al., 2016). At‐homeness is relevant across settings and previously identified as a concept relevant to in relation to care home transitions, in addition to other transitions (Holland et al., 2017; Roy et al., 2018; Saarnio et al., 2018). The at‐homeness facet of being oneself relates to being able to manage one's everyday life. For some residents in our study, this control over everyday life was disrupted by the main decision by the managing organisation that relocation was needed. Residents and family members welcomed the choices they were given about room location and decor but the new environment also limited control in important ways. Some residents struggled with the larger size and layout of the physical environment and this reduced their freedom to move around at will and take part in activities outside of their rooms, thus impacting the at‐homeness of their experience in the new home. Therefore, we suggest that staff, residents and their families should be involved in the planning and design of the new home as these issues might have been picked up in advance.

At‐homeness also relates to being connected with other people. This means being close to significant others, being in affirming friendships and being in safe dependency (Saarnio et al., 2016). Relationships from the original tight‐knit community, described as like a ‘family’ were disrupted by the move. Residents had not usually considered their room location choice in relation to being near to their fellow residents who were also friends. The challenging size and layout constrained some people from either visiting each other or meeting up in communal spaces. In addition, the increased size of the new home and the accompanying need to increase staff numbers meant that residents were often not in receipt of care from individual staff that they were familiar with. This had a negative impact on residents’ adjustment to the new home and highlights the need to consider staffing to support continuity of care in future relocations as suggested in Box 1. Our findings also suggest that the relocation resulted in diminished feelings of at‐homeness for some residents, in spite of significant efforts by senior managers to provide a high‐quality environment. Residents and relatives should be offered the chance to visit the new care home to assess where their room will be in relation to other facilities and consider room choices near to their friends.

A focus on being connected with other people through relationships and the well‐being of all participants in care environments for older people underpin the delivery of high‐quality care (Nolan et al., 2006). Our findings draw attention to the experiences of staff members and family members, as well as residents, and the needs of all to feel connected with others. Staff and family member interviews highlight inconsistencies in perceived levels of their involvement in decisions about the new home, and that some who felt left out of decisions perceived that the experiences of residents had been compromised. Care home cultures where older people, their families and staff are supported to develop positive relationships with one another (relationship‐centred care) enable staff to connect with residents and respond to them as individuals, and facilitate resident choice and control (Nolan et al., 2006). Enabling residents to have voice, choice and control is particularly important during transitions in care (Owen & Meyer, 2012), such as care home relocations, and our findings draw attention to the complexities of exactly how shared decision‐making can be achieved when a complex project has multiple stakeholders, each with different preferences and expectations about the new facility. The findings add a new dimension to the at‐homeness literature, demonstrating the relevance of the concept to whole care home transitions, and highlighting family and staff experiences, as well as residents. The findings also emphasise the work needed and the time it takes to build a new community in tandem with a move to a new, larger site. Our findings of diminished experiences up to 12 months after the physical move suggest that the need for work on building the care home community should extend beyond the planning stage and the move itself.

We were able to draw recommendations and guidance that could be helpful not only for similar whole care home relocations in the future but also to any individual care home relocation. These recommendations should be used alongside the guidance published by Jolley et al. (2011). Their guidance provides general recommendations for relocation including good communication with and involvement of all those who are key in the relocation, performing health checks on residents pre and post the move, and good and regular documentation of any communications, checks or handover notes. Our guidance (see Box 1) is drawn from the lived experience of a relocation over a prolonged period of time and focuses on providing recommendations helpful for group relocation of staff and residents together not only to a newly built care home but also to care home relocation in general. For example, an at‐homeness feeling, continuity of care and consistent communication are important issues that should be considered in any care home relocation. We also draw some recommendations more specific for relocations to new care homes such as timing of familiarisation visits, involvement of staff, residents and family members in the design of the new care home.

Our study had a number of strengths with longitudinal data collection and inclusion of different stakeholders (residents, family members and staff) of the care home community, which provides a relatively novel approach to research of this kind. However, it was a small study based on one care home relocation, and a number of participants did not take part in the follow‐up interviews. The 12 months follow‐up period may have included physical and cognitive deterioration in the residents which could have impacted on their views and experience in the new care home. Residents interviewed were all female and they had been in the old care home for varying lengths of time; however, data on the exact residents’ length of stay were not collected. We interviewed staff at junior, senior and managerial levels but did not record how long they had worked in the care home, and thus, were unable to explore whether these factors influenced the experience of home relocation. We recommend these characteristics are collected in future research. We have followed a direct approach to recruit participants to the study which might have introduced a sampling bias. However, we had used a number of strategies to mitigate any bias including allowing enough time to participants to decide whether to take part or not, and approaching participants by research team instead of the care home management team or staff member to avoid any pressure. We were unable to collect reasons for non‐participation among those who were eligible to join the study but chose not to take part. It is possible that these individual residents, family members and staff had different opinions and experience to those expressed by individuals interviewed. However, the interviews conducted clearly provide space for participants to acknowledge dissatisfaction, desire for some things to be different, and were not all complimentary. Thus, we feel that the study data remains an important source for recommendations for future relocations. Furthermore, ideally participants should also have been interviewed before the relocation to understand their expectations, preparation and attitudes towards the move. There is the possibility that the reflection on their experience after the relocation may have influenced their account expressed during the interviews.

## 5 CONCLUSIONS

This study reports the experience of residents, family members and staff of a planned whole care home relocation over 1 year. Despite implementation of existing guidance regarding planned relocation, moving and adjustment was still challenging for some participants although time, continuity of care and family support was helpful. Our findings also suggest that at‐homeness feelings and maintaining social relationships among residents are important factors that should be considered in planning any future relocation. The new environment including its physical size and layout as well as staff organisation and management were identified as important factors for consideration. We have developed recommendations to support future whole care home relocations to help guide current policy and practice.

## CONFLICT OF INTEREST

KI, JL, SB and NM declare no conflicts of interest. HR and JB were trustees of the charitable foundation responsible for the care homes when the data were gathered.

## AUTHOR CONTRIBUTIONS

All authors contributed to the conception and design of the study and developing and approving the final paper. KI, JL and SB contributed to data collection and analysis, KI, JL, SB and JB drafted the manuscript.

## Data Availability

Due to the nature of this research, participants of this study did not agree for their data to be shared publicly, so supporting data are not available.
